# Growth Parameters and Prevalence of Obesity in PKU Patients and Peers: Is This the Right Comparison?

**DOI:** 10.3390/pediatric16040076

**Published:** 2024-10-16

**Authors:** Giulia Paterno, Vito Di Tullio, Rosa Carella, Giada De Ruvo, Fabrizio Furioso, Aleksandra Skublewska-D’Elia, Donatella De Giovanni, Albina Tummolo

**Affiliations:** 1 Department of Metabolic Diseases, Clinical Genetics and Diabetology, Giovanni XXIII Children Hospital, Azienda Ospedaliero-Universitaria Consorziale, 70126 Bari, Italy; giulia.paterno@policlinico.ba.it (G.P.); vditullio11@libero.it (V.D.T.); rosa.carella@policlinico.ba.it (R.C.); f.furioso1@studenti.uniba.it (F.F.); donatella.degiovanni@policlinico.ba.it (D.D.G.); 2 Vivenda SPA, Strada Torre Tresca, 18, 70124 Bari, Italy; giada.deruvo@vivendaspa.it; 3 General Pediatrics, Local Health Authority, 70126 Bari, Italy; aleksandra.skublewska@gmail.com

**Keywords:** phenylketonuria, growth, body mass index, overweight, obesity

## Abstract

Background: One of the main objectives of Phenylketonuria (PKU) management is represented by optimising the growth trend under restricted protein diet regimen. The data on long-term growth in PKU children are limited and mostly based on earlier studies. Methods: The data for this twelve-year longitudinal study were collected from 34 PKU children and 37 healthy peers, whose auxological parameters were taken at 7 time-points over the follow-up. The weight-for-length ratio (WLR) z-score and body mass index (BMI) z-score were considered according to age. Prevalence of overweight/obesity was evaluated at last assessment. Results: The median BMI z-score of PKU children was normal and not statistically different from that of controls on all the seven time-point assessments. Their distributions tended to be wider than those of peers, with the upper limit exceeding the normal range since 12 months old, with a peak specifically at 3 years of age. In controls, there was a tendency to approach the BMI z-score values of overweight in later childhood. The prevalence of overweight was comparable (29% vs. 25%, *p*: 0.78) between the two groups at last assessment, and obese subjects (3/37) were only detected in the control group. Conclusions: In this study, we report data from a long-term follow-up on growth, highlighting that the median BMI z-score of PKU children was normal and not statistically different from that of controls. Also, the prevalence of obesity at 12 years of age was overlapping. However, the high prevalence of overweight children in the general population may explain the lack of difference and does not reassure about patients’ nutritional risk.

## 1. Introduction

Phenylketonuria (PKU) is an inherited disease characterised by high levels of phenylalanine (Phe) in plasma due to an abnormal activity of Phe hydroxylase (PAH), which converts Phe into Tyrosine [1]. If left untreated, high Phe blood levels can cause neurological damage leading to intellectual disability, epilepsy as well as behavioural, psychiatric and movement abnormalities [2].

Early diagnosis has been possible for many years, thanks to newborn screening programmes developed worldwide, with the primary aim to keep blood Phe levels within a therapeutic target range from the first days of life [3].

Diet therapy is the main treatment approach to PKU [4]. Indeed, an appropriate and prompt dietary intervention allows affected individuals to avoid disease complications and to improve outcomes [5]. Dietetic management is based on restriction of Phe intake by avoiding or reducing natural protein food, particularly food rich in animal proteins [6], according to individual Phe tolerance. Energy intake is guaranteed by fruits and vegetables, which are naturally poor in Phe content, but also by sugars, fats and oils [7].

Special low-protein foods and Phe-free amino acids (L-AAs) or low-Phe glycomacropeptide supplemented with aminoacids (GMP-AA) complete the dietetic scheme of PKU diets, ensuring appropriate protein and caloric intake and avoiding catabolism [8,9]. There is nowadays a wide range of protein substitute options available, offering individuals with PKU more choices to suit their preferences and dietary needs. However, while much emphasis has been given to the amino acids content of these medical foods, less attention is classically given to the nutritional and energy profile and fat content of such supplements [10].

The unbalanced diet composition and the more frequent use of industrial products, compared to the normal population’s diet, raise attention about possible effects on patients’ nutritional status, particularly on growth trends in paediatric age [11,12,13]. In this context, overweight and obesity tendencies in PKU children have been reported by many authors [14,15,16,17,18], although the difference with the general population and the trend over the developmental period are still inconclusive and need further investigation.

The aim of this study is to compare the growth trend of PKU children over the first twelve years of life with control peers and to assess the prevalence of overweight/obesity at the last clinical assessment.

## 2. Materials and Methods

### 2.1. Patients

The data for this longitudinal case-control study were collected from April to July 2023. A total of 40 PKU subjects and 40 controls were enroled. The inclusion criteria for PKU patients were diagnosis through neonatal screening, regular follow-up since birth at the referral centre, administration of a diet with reduced Phe intake, availability of height and weight at the clinical follow-up visits since birth and Phe levels falling within the target levels for at least 75% of all the measured values (intended to be as good dietary compliance).

The controls enroled were among those who attended the general paediatrician and who were followed through regular visits since birth until 12 years of age. The exclusion criteria were irregular follow-up (more than two years loss-of-follow-up) and/or presence of severe comorbidities, such as serious chronic cardiovascular, haematological, endocrine renal, hepatic, neurological, gastrointestinal, rheumatic and skeletal conditions, severe infections and any other illness that could severely influence nutritional status.

PKU was defined as classic if the screening Phe level was >1200 µmol/L and mild/moderate if it fell between 600 and 1200 µmol/L. Target levels were 120–360 µmol/L, according to the European Guidelines [7].

### 2.2. Measurements

For the recruited subjects, the auxological parameters were taken at 7 time-points over the follow-up, and, more specifically at 0, 6 and 12 months and 3, 6, 9 and 12 years. Growth parameters were detected by two expert paediatricians: one for PKU patients and the other for healthy subjects.

For patients younger than one year, the digital paediatric balance Wunder BABY 01^®^ (Milano, Italy) and Seca Infantometer foldable 417^®^ (Hamburg, Germany) were used. For patients older than 1 year, a Seca wall-mounted stadiometer ^®^ (Hamburg, Germany) and a Tanita BC 418 MA 3 Body Composition Analyzer ^®^ (Tokyo, Japan) were used.

Body weight, length and head circumference were measured and recorded. Weight-for-length ratio (WLR) was calculated up to twelve months of life, and body mass index (BMI) was instead used from 1 year up to 12 years of age. Z-scores were calculated at the seven time-points for both groups, referring to CDC Growth Charts [19,20]. Self-reported paternal and maternal weights and heights were also recorded for both groups.

BMI was calculated as the weight/height^2^ ratio, and normal values of the BMI z-score ranged between +2.0 and −2.0 according to the World Health Organization (WHO) criteria charts [21]. Participants were then classified into four BMI categories based on the World Health Organization (WHO) criteria [16]: group 1: underweight (BMI standard deviation (SD) score of −5 or more but less than −2); group 2: normal weight (BMI SD score of −2 or more but less than 1); group 3: overweight (BMI SD score of 1 or more but less than 2) and group 4: obese (BMI SD score of 2 or more but less than 5). The last assessment, at 12 years of age, was considered the final auxological framework.

Macronutrients and caloric intakes derived from medical foods and natural foods and based on 3-day food records were reported. Food record analyses were conducted by a dietitian experienced in inherited metabolic disorder diets. Winfood Pro software (version 3.0.0, 2011, Medimatica Srl, Teramo, Italy) was used for intakes calculation. Energy intake was expressed as kcal/day and compared with FAO/WHO/UNU 2005 requirements [22]. Patients’ way of lactation (breastfeeding and/or milk formulas), as well as time and modalities of weaning, were recorded for both patients and healthy peers.

The dietary management of patients with PKU involved the use of Phe-free amino acid mixtures, possibly with the addition of breast milk, where available, during the first six months of life. Alternatively, formula milk was used [7]. Starting from six months of age, weaning was commenced with the gradual introduction of low-protein foods, such as low-protein semolina or pasta and vegetables.

For the control group, the guidelines of the Italian Society of Preventive and Social Pediatrics [23] were followed for the first six months for lactation and for nutrition since weaning onwards.

All dietary and auxological procedures were in agreement with the guidelines of the Declaration of Helsinki on Human Experimentation; this study was based on standard clinical practice and did not involve any additional procedures or interventions; therefore, it did not require approval by the Local Ethics Committee.

### 2.3. Statistics

Statistical analysis was carried out using SPSS 15.0 for Windows (SPSS Inc., Chicago, IL, USA). Results were considered as statistically significant when *p* < 0.05. Data which were normally distributed were presented as means and standard deviations (SDs), and not normally distributed data were instead presented as median and 25th–75th interquartile range (IQR). Differences between the study groups were assessed using independent *t*-tests or the Chi^2^ test as appropriate.

Data were analysed for repeated measures using ANOVA or the Kruskal–Wallis test according to the variables’ type of distribution. Post hoc comparisons with Bonferroni’s correction were used to test for specific comparisons between time-points and between the two groups at each time-point.

## 3. Results

### 3.1. Participants

Out of 40 children with PKU, 34 (85.1%) took part in this study. Insufficient data (4) and unwillingness to participate (2) led to the exclusion of patients from the study. Among controls, 3 out of 40 were excluded (7.5%) because they were twins with VLBW (weight < 1500 g). Finally, 71 participants were included and analysed in this study.

### 3.2. Demographic Characteristics

The demographic and perinatal data are reported in Table 1. Among patients, those with classic PKU consisted of 17/34 (50%), and those with mild/moderate PKU were the remaining 50%. The z-score of neonatal weight and height did not differ significantly between patients and controls. There were also no significant differences between the parents’ auxological parameters, except for maternal height, which was greater in the control group than in the PKU group (*p*: 0.02). All patients were Caucasian, with a higher prevalence of males, although not statistically higher than female gender (Table 1).

### 3.3. BMI Z-Score Changes over Time

**Figure 1:**
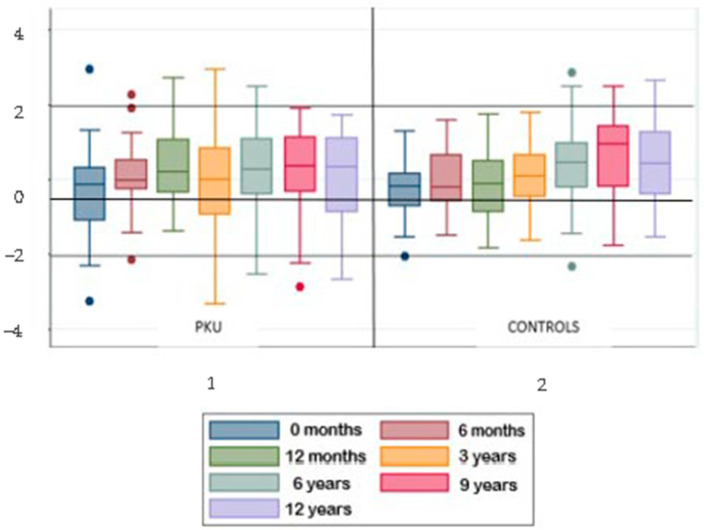
BMI z-score trends at the 7 time-points for patients and controls.

Medians and interquartile ranges were assessed at the seven follow-up time-points for BMI z-scores of both patients and controls. Figure 1 reports the box-plots of their distribution. The median BMI z-score of PKU children at different time-points remained almost stable around 0 throughout childhood. However, the ranges were quite wide, exceeding the higher or lower limits of −2 and +2 at all observations but that at 6 months of age. The ranges of BMI z-scores at the 3 years observation were particularly wide, with both upper and lower limits exceeding the +2/−2 range.

In controls, the median remained also stable around 0, with extremes of distribution within the interval of +2 and −2 for all the observations until 3 years of age. Starting from 6 years of age on, the median tended to deviate from 0 upwards, reaching +1 SD at 9 years of life. In addition, from the age of six, the upper limit of the distribution stably exceeded +2 SD. The comparison among medians using the Kruskal–Wallis test showed no significant differences of BMI z-score at the different time-points between patients and controls (*p*-value: 0.12).

### 3.4. Dietary Intake of PKU Patients at Follow-Up Visits

Overall, 13 out of 34 (38%) PKU patients were breastfed during the first six months of life, whereas the other 21 (62%) consumed milk formulas, in both cases, in association with Phe-free formulas. In all cases, weaning was commenced between five months and a half and six months of age, starting from the same proteic intake of the lactation time.

In the control group, 22 out of 37 (60%) were breastfed for the first six months of life, 9 (24%) received a mix feeding and 6 (16%) were only fed with milk formulas since birth. Weaning was started at five months of age for 13 children (35%), and at six months of age for the other 24 (65%).

According to FAO/WHO/UNU recommendations [22], a gradual increase in energy and Phe intake according to age was performed. Data of intakes at the 7 timepoints are reported in Table 2. The degrees of increase were calculated on the basis of what was reported by the official guidelines [7], but they were always personalised on the basis of growth trends and Phe tolerance. The consumption of aproteic food was always included within the total amount of protein intake, and their amount was modulated also on the basis of the target caloric content, as they represented the main source of carbohydrates and fats.

### 3.5. Data at the Last Observation

The majority of subjects in both groups had a BMI z-score within the normal range: 57% for both groups (*p* = 0.95).

**Figure 2:**
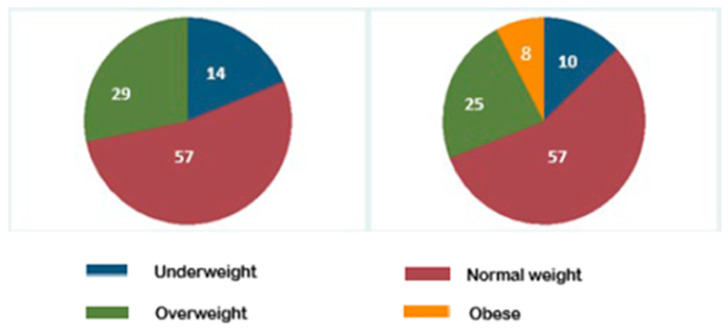
Pie charts on BMI z-score category percentage at last assessment.

The prevalence of subjects in the overweight category in the PKU group was 29% (10/34 (Figure 2)), and no patient had a BMI z-score in the range of obesity. Only one patient at the 12-year assessment had a BMI z-score in the underweight range. This patient was born at 30 weeks of gestational age, with a weight of 1.30 kg (z-score—3.03) and a length of 40 cm (z-score—3.66) below the normal range. Its growth trend persisted in the lowest percentile for both weight and height all throughout the follow-up.

In the control group, 9 out of 37 (25%) subjects were in the overweight category, whereas 3 out of 37 subjects resulted to be obese (8%) (Figure 2). Considering the distribution of the BMI z-score categories by sex, there was no significant difference in the prevalence of male sex in the overweight subgroup compared to females (28% vs. 24%, *p*: 0.71). The three subjects in the obesity range were all males.

There were no statistically significant differences in caloric and Phe contents at the last assessment between normal weight and overweight children (Table 3).

The multivariable linear regression did not highlight a correlation between BMI z-score values at 12 years and sex, birth weight and maternal and paternal BMI.

## 4. Discussion

Since newborn screening has reduced the incidence of neurological sequelae linked to PKU, one of the main objectives of PKU management is represented by optimising the growth trend under a restricted protein diet regimen [4].

In this study, we report data from a long-period follow-up on growth, highlighting that the median BMI z-scores of PKU children were normal and not statistically different from those of controls during the first twelve years of life. Their distributions tended to be wider than those of controls, with the upper limit exceeding the normal limit starting at 12 months and with a peak at 3 years of age. In controls, there was a tendency to approach the BMI z-score values of overweight in later childhood. The prevalence of overweight was comparable between the two groups at the last assessment, and no obese subjects were detected in the PKU group.

Data on long-term growth in PKU patients are scarce and mostly based on earlier studies which do not include the recent products available for PKU diets. Indeed, over the last 10 years, the dietary therapy approach for PKU has evolved significantly, with more advanced medical foods being developed, offering improved protein substitutes with better taste profiles and higher acceptability.

If, from the one side, the natural proteins consumed every day by this category of patients mainly come from fruit and vegetables, which have a lower protein availability than foods of animal origin and also a low caloric content, on the other, the free consumption of industrial products with low Phe content may consistently increase the daily caloric intake.

Various studies have analysed the prevalence of overweight and obesity among PKU patients, comparing them with healthy peers. Gokmen et al. [17] showed that there is no difference in the tendency to develop overweight/obesity between the PKU patient population and the healthy population. Rocha et al. reported that the PKU population tends to develop overweight between 20 and 30%, while the obese population has a prevalence lower than 10%, mainly after 12 years of age [24].

In our study, the trend to gain weight in PKU children starts earlier than in healthy peers and can be attributable at least partially to the composition of many foods that these subjects eat. The protein:energy (P:E) ratio of protein substitutes is lower in the starting formulas than in subsequent formulas; therefore, in the 0–4 years age group, children undergo an increased caloric intake in order to reach the daily protein requirement [25,26]. Furthermore, there was a higher proportion of milk-formula-fed infants among patients than in controls, which has been classically associated with excess or rapid weight gain [27].

All the above can at least partially explain the higher trend found in our study toward being overweight in the first three years of life.

Excessive caloric intake may be also determined by special low-protein food (SLPF), the consumption of which can be free, as it does not influence Phe values. Comparison between the composition of SPLF and regular food shows that there is a trend of higher saturated fat content, palm oil and hydrogenated vegetable oil and sugar and carbohydrate content, which determines a higher energy content than natural food [11,28], although not significantly altering their glycaemic index, because of a greater amount of fibre in SPLF compared to normal foods [29].

A better composition of amino acid mixtures and SLPFs could certainly help improve the quality of nutrition in these patients.

A retrospective study [10] based on data relatd to pharmaceutical expenditure has reported that the incidence of diabetes mellitus, arterial hypertension and dyslipidaemia is significantly higher in adult PKU patients than in the general population. These lines of evidence raise the importance of detecting early risk factors in this particular group of patients since the paediatric age.

The tendency towards overweight and obesity since childhood has been widely associated with the increased risk of cardiovascular disease in the general population [30]. According to International Obesity Task Force (IOTF) data, the percentage of overweight in the healthy population is around 25% in children and adolescents (12–19 years) and 30–40% in adults, which increases over the years [31].

In our population, the prevalence of overweight remained around 25% also over 12 years (data not shown), corroborating the hypothesis that the growth trend during childhood is paramount for later BMI [5,32].

The high incidence of overweight/obesity in the general population is mainly related to an unbalanced diet, characterised by high consumption of sugary drinks and fast food, associated with a reduced intake of fruit and vegetables and low physical activity; this tendency is higher as children approach adolescence [33]. In our control sample, a trend toward overweight was, in fact, particularly evident after six years of age and while approaching adolescence.

This study has some strengths and limitations. It provides long-term longitudinal mirroring of the current dietary practices, whereas most growth data come from studies on children with PKU who were born before 1990 [34]. The main limitation comes from the numerosity of the sample size and from the lack of information on real (and not prescribed) nutritional intake, which limited the possibility of a correlation of the BMI z-score with metabolic control. Indeed, it is likely that, in a diet-controlled disease, such as PKU, the compliance with the prescribed diet may not be complete. However, we have tried to minimise this issue by including only those patients with satisfactory metabolic control for whom a good dietetic compliance could be assumed.

Also, in this study, the role of physical activity as a cofactor of the observed z-score BMI was not investigated, although, in a recent study from our group on hyperphenylalaninemia children [35], we found that only 4% of patients performed active physical activity versus 38% of controls, who also demonstrated a better acceptance of body image compared to patients.

The diet of PKU patients has changed over time and tends to be as close as possible to that of the general population. The high trend toward a sedentary lifestyle represents a social problem involving also subjects undergoing a special diet regimen [36].

The question on the basis of this study derives from the broader ongoing debate about the best way to judge the nutritional status of children, as body size is determined by several factors: genetic; hormonal; nutrition; parental characteristics; socioeconomic status [37]. The comparison with the global standard measurement or with the paired population growth trend can therefore result in an inaccurate interpretation and classification of the growth trend. The analysis of single determinants of patients’ health status may result in a better everyday practice and a more precise decision making for all those involved in children’s nutritional management. Therefore, the lack of difference in growth and nutritional parameters between the two groups does not reassure about patients’ nutritional risk. Strategies to ameliorate nutritional patterns in PKU children should include both improvement in medical food composition and educational intervention common to those of the general population.

## Figures and Tables

**Table 1:** Main auxological characteristics of study participants.

	PKU Patients (34)	Controls (37)	*p*-Value
Gender *n* (%)	14 F/20 M (41/59)	12 F/28 M (30/70)	0.44
Type of PKU *n* (%)	17 classic (50%) 17 mild/moderate (50%)	-	0.92
Birth weight z-score	−0.22 (−0.59–0.32)	−0.45 (−0.81–0.04)	0.19
Birth height z-score	0.33 (−0.12–0.76)	−0.12 (−0.37–0.38)	0.39
Birth w/L z-score	−0.12 (−1.87–0.32)	−0.19 (−0.72–0.17)	0.91
Mother weight (kg)	60 (54–73)	61.5 (58–70)	0.97
Mother height (cm)	161 (157–165)	165 (160.5–170)	**0.02**
Mother BMI (kg/m^2^)	24.1 (21–29)	22.7 (20.4–24.2)	0.13
Father weight (kg)	78 (73–90)	78 (71–84)	0.66
Father height (cm)	175 (170–182)	175 (171–180)	0.92
Father BMI (kg/m^2^)	25.7 (23.3–28.3)	25.2 (23.6–26.2)	0.5

Median (interquartile range) for all quantitative variables.

**Table 2:** Dietary intakes (median (interquartile range)) of PKU patients at the 6 time-points.

Intakes	6 Months	12 Months	3 Years	6 Years	9 Years	12 Years
Energy (kcal)	846 (761–921)	1078 (1030–1138)	1401 (1304–1467)	1682 (1603–1769)	1854 (1767–1966)	2009 (1850–2161)
Energy kcal/kg	107 (99–115)	105 (98–114)	93 (88–101)	81 (68–87)	62 (47–72)	51 (42–58)
Phe (mg/day)	285 (240–345)	317 (271–390)	327 (300–410)	348 (305–460)	351 (305–500)	373 (305–510)
Phe (mg/kg/day)	38 (33–56)	32 (26–37)	23 (20–32)	19 (14–23)	12 (10–18)	10 (7–14)

**Table 3:** Dietary intake (mean ± SD) of PKU patients totally and according to BMI z-score category at last assessment.

Intakes at Last Assessment	All Patients	Normal Weight	Overweight/Obese	*p*-Value
Energy (kcal)	2009 (1850–2161)	2070 (1924–2171)	1825 (1720–1930)	0.08
Phe (mg/day)	373.5 (305–510)	388 (305–510)	365 (285–635)	0.82
Phe (mg/kg/day)	9.6 (7.4–14.3)	9.6 (8.1–15.1)	7.7 (5.3–14.3)	0.50

## Data Availability

The data presented in this study are available on request from the corresponding author.

## References

[1] Elhawary N.A., AlJahdali I.A., Abumansour I.S., Elhawary E.N., Gaboon N., Dandini M., Madkhali A., Alosaimi W., Alzahrani A., Aljohani F. (2022). Genetic etiology and clinical challenges of phenylketonuria. Hum. Genom..

[2] Brown C.S., Lichter-Konecki U. (2016). Phenylketonuria (PKU): A problem solved?. Mol. Genet. Metab. Rep..

[3] Blau N., Hennermann J.B., Langenbeck U., Lichter-Konecki U. (2016). Diagnosis, classification, and genetics of phenylketonuria and tetrahydrobiopterin (BH4) deficiencies. Mol. Genet. Metab..

[4] Blau N., Bélanger-Quintana A., Demirkol M., Feillet F., Giovannini M., MacDonald A., Trefz F.K., Van Spronsen F. (2010). Management of phenylketonuria in Europe: Survey results from 19 countries. Mol. Genet. Metab..

[5] Rocha J.C., MacDonald A. (2016). Dietary intervention in the management of phenylketonuria: Current perspectives. Pediatr. Health Med. Ther..

[6] Rohde C., Mütze U., Weigel J.F.W., Ceglarek U., Thiery J., Kiess W., Beblo S. (2012). Unrestricted consumption of fruits and vegetables in phenylketonuria: No major impact on metabolic control. Eur. J. Clin. Nutr..

[7] Van Wegberg A.M.J., Macdonald A., Ahring K., BéLanger-Quintana A., Blau N., Bosch A.M., Burlina A., Campistol J., Feillet F., Giżewska M. (2017). The complete European guidelines on phenylketonuria: Diagnosis and treatment. Orphanet J. Rare Dis..

[8] Daly A., Pinto A., Evans S., MacDonald A. (2022). Glycomacropeptide in PKU-Does It Live Up to Its Potential?. Nutrients.

[9] Daly A., Högler W., Crabtree N., Shaw N., Evans S., Pinto A., Jackson R., Strauss B.J., Wilcox G., Rocha J.C. (2021). Growth and Body Composition in PKU Children-A Three-Year Prospective Study Comparing the Effects of L-Amino Acid to Glycomacropeptide Protein Substitutes. Nutrients.

[10] Pena M.J., Almeida M.F., van Dam E., Ahring K., Bélanger-Quintana A., Dokoupil K., Gokmen-Ozel H., Lammardo A.M., MacDonald A., Robert M. (2015). Special low protein foods for phenylketonuria: Availability in Europe and an examination of their nutritional profile. Orphanet J. Rare Dis..

[11] Evans S., Daly A., Wildgoose J., Cochrane B., Chahal S., Ashmore C., Loveridge N., MacDonald A. (2019). Growth, Protein and Energy Intake in Children with PKU Taking a Weaning Protein Substitute in the First Two Years of Life: A Case-Control Study. Nutrients.

[12] Thiele A.G., Gausche R., Lindenberg C., Beger C., Arelin M., Rohde C., Mütze U., Weigel J.F., Mohnike K., Baerwald C. (2017). Growth and Final Height Among Children With Phenylketonuria. Pediatrics.

[13] Matic J., Zeltner N.A., Häberle J. (2019). Normal Growth in PKU Patients Under Low-Protein Diet in a Single-Center Cross-Sectional Study. JIMD Rep..

[14] Verduci E., Banderali G., Moretti F., Lassandro C., Cefalo G., Radaelli G., Salvatici E., Giovannini M. (2015). Diet in children with phenylketonuria and risk of cardiovascular disease: A narrative overview. Nutr. Metab. Cardiovasc. Dis..

[15] Rocha J.C., MacDonald A., Trefz F. (2013). Is overweight an issue in phenylketonuria?. Mol. Genet. Metab..

[16] Yılmaz B.K., Baykan A., Kardaş F., Kendirci M. (2023). Evaluation of the effect of obesity, dietary glycemic index and metabolic profiles on the cardiovascular risk in children with classical phenylketonuria. Mol. Genet. Metab..

[17] Gokmen H., Ozel K.A., Bélanger-Quintana A. (2014). Overweight and obesity in PKU: Results from 8 centres in Europe and Turkey. Mol. Genet. Metab. Rep..

[18] Ahmadzadeh M., Sohrab G., Alaei M., Eini-Zinab H., Mohammadpour-Ahranjani B., Rastgoo S., Namkhah Z. (2022). Growth and Nutritional Status of Phenylketonuric Children and Adolescents. BMC Pediatr..

[19] Chou J.H., Roumiantsev S., Singh R. (2020). PediTools Electronic Growth Chart Calculators: Applications in Clinical Care, Research, and Quality Improvement. J. Med. Internet Res..

[20] Flegal K.M., Cole T.J. (2013). Construction of LMS parameters for the Centers for Disease Control and Prevention 2000 growth chart. Natl. Heal. Stat. Rep..

[21] Cole T.J., Lobstein T. (2012). Extended internation (IOTF) body mass index cut-off for thinness, overweight and obesity. Pediatr. Obes..

[22] Joint F.A.O. (2005). Human energy requirements: Report of a joint FAO/WHO/UNU Expert Consultation. Food Nutr. Bull..

[23] Cuzzolin M. (2016). Manuale di Nutrizione in età Evolutiva.

[24] Rocha J.C., van Spronsen F.J., Almeida M.F., Soares G., Quelhas D., Ramos E., Guimarães J.T., Borges N. (2012). Dietary treatment in phenylketonuria does not lead to increased risk of obesity or metabolic syndrome. Mol. Genet. Metab..

[25] Gomes M., Almeida M.F., Barbosa C.S., Gama M.I., Peres M., Pinto É., MacDonald A., Rocha J.C. (2023). Total Protein Intake in Patients with PKU: Adequacy Evaluation According to the European PKU Guidelines from 2017. Nutrients.

[26] McWhorter N., Ndugga-Kabuye M.K., Puurunen M., Ernst S.L. (2022). Complications of the Low Phenylalanine Diet for Patients with Phenylketonuria and the Benefits of Increased Natural Protein. Nutrients.

[27] Appleton J., Russell C.G., Laws R., Fowler C., Campbell K., Denney-Wilson E. (2018). Infant formula feeding practices associated with rapid weight gain: A systematic review. Matern. Child Nutr..

[28] Wood G., Evans S., Pointon-Bell K., Rocha J.C., MacDonald A. (2020). Special Low Protein Foods in the UK: An Examination of Their Macronutrient Composition in Comparison to Regular Foods. Nutrients.

[29] Garcia-Arenas D., Barrau-Martinez B., Gonzalez-Rodriguez A., Llorach R., Campistol-Plana J., García-Cazorla A., Ormazabal A., Urpi-Sarda M. (2023). Effect of Special Low-Protein Foods Consumption in the Dietary Pattern and Biochemical Profile of Patients with Inborn Errors of Protein Metabolism: Application of a Database of Special Low-Protein Foods. Nutrients.

[30] Zong X., Kelishadi R., Kim H.S., Schwandt P., Matsha T.E., Mill J.G., Whincup P.H., Pacifico L., López-Bermejo A., Caserta C.A. (2024). Utility of waist-to-height ratio, waist circumference and body mass index in predicting clustered cardiometabolic risk factors and subclinical vascular phenotypes in children and adolescents: A pooled analysis of individual data from 14 countries. Diabetes Metab. Syndr. Clin. Res. Rev..

[31] Engin A. (2017). The Definition and Prevalence of Obesity and Metabolic Syndrome. Adv. Experimantal Med. Biol..

[32] Halilagic A., Moschonis G. (2021). The Effect of Growth Rate during Infancy on the Risk of Developing Obesity in Childhood: A Systematic Literature Review. Nutrients.

[33] Jakobsen D.D., Brader L., Bruun J.M. (2023). Association between Food, Beverages and Overweight/Obesity in Children and Adolescents—A Systematic Review and Meta-Analysis of Observational Studies. Nutrients.

[34] Ilgaz F., Pinto A., Gökmen-Özel H., Rocha J.C., van Dam E., Ahring K., Bélanger-Quintana A., Dokoupil K., Karabulut E., MacDonald A. (2019). Long-Term Growth in Phenylketonuria: A Systematic Review and Meta-Analysis. Nutrients.

[35] Dicintio A., Paterno G., Carella R., Ortolani F., Masciopinto M., De Giovanni D., Tummolo A. (2022). Food Habits and Lifestyle in Hyperphenylalaninemia Patients: Should These Be Monitored?. Children.

[36] Jani R., Coakley K., Douglas T., Singh R. (2017). Protein intake and physical activity are associated with body composition in individuals with phenylalanine hydroxylase deficiency. Mol. Genet. Metab..

[37] Sachdev H.S., Borghi E. (2024). Should a single growth standard be used to judge the nutritional status of children under age 5 years globally? No. Am. J. Clin. Nutr..

